# Relationship between male aging and semen quality: a retrospective study on over 2500 men

**DOI:** 10.1007/s00404-024-07448-8

**Published:** 2024-03-29

**Authors:** Chiara Castellini, Giuliana Cordeschi, Daniele Tienforti, Arcangelo Barbonetti

**Affiliations:** Andrology Unit, Department of Clinical Medicine, Life, Health and Environmental Sciences, University of L’Aquila, Coppito, 67100 L’Aquila, Italy

**Keywords:** Ageing, Infertility, Oxidative stress, Sperm morphology, Sperm motility, Spermatozoa

## Abstract

**Purpose:**

We aimed to evaluate whether and to what extent an association exists between male aging and worsening of semen parameters and to determine whether a threshold age can be identified above which the decline in semen quality becomes statistically significant.

**Methods:**

2612 men (age: 16–56 years) attending an andrology outpatient clinic for semen analysis and clinical evaluation were studied. Semen analyses were performed according to the ongoing WHO-recommended procedures. Total motile count (TMC) and total progressive motile count (TPMC) were calculated by multiplying total sperm number by total motility and progressive motility, respectively.

**Results:**

Significant negative correlations were found between age and total motility (*r* = − 0.131, *p* < 0.0001), progressive motility (*r* = − 0.112, *p* < 0.0001), TPMC (*r* = − 0.042, *p* = 0.037), and normal sperm morphology (*r* = − 0.053, *p* = 0.007). All these associations persisted in multivariate regression models adjusted for abstinence time, smoking, history of male accessory gland infections, varicocele and the year in which semen analysis was performed. When comparisons were performed among quartiles of increasing age, the fourth quartile, corresponding to the age group > 40 years, was associated with a significant decrease in total and progressive motility. An earlier decline in the TPMC and percentage of normal forms was also observed.

**Conclusion:**

Advancing male age exhibits an independent association with a decrease in the percentage of motile and morphologically normal spermatozoa, with greater evidence from the age of > 40 years. Further studies are warranted to elucidate the mechanisms and clinical reflections of these associations.

## What does this study add to the clinical work

**Table:** 

Advancing male age is associated with a decrease in the proportion of motile and morphologically normal spermatozoa, independent of a number of confounding clinical variables and with greater evidence from the age above 40 years. Healthcare providers should be aware that worsening seminal quality with age can impair the male partner's ability to compensate for a female subfertility condition resulting in an overall weakening of the couple's reproductive potential.	

## Introduction

While it is well documented that advanced female age is associated with worse couple fertility and reproductive outcomes, information regarding the impact of aging on male reproductive potential remains controversial, although increasingly advanced paternal age is raising concern in recent years. Trends in the average age of parenthood in Western countries over the past three decades revealed a gradual decrease in number of births within couples with male partners aged < 35 years, with a drop between the ages of 25 and 30 years, and a concomitant increase in those associated with paternal age in the range from 35 to 44 years [1]. In a large U.S. study that collected information on nearly 170 million births from the databases of the Centers for Disease Control, over the 40-year period from 1972 to 2015, the increasing trend in the age of parenthood covered all ethnic groups, rising overall from an average age of 27–31 years [2]. On this basis, today, in the U.S., 9–10% of new births have fathers over 40 years, a percentage that has virtually doubled since the 1980s. Beyond the socioeconomic and epidemiological reasons behind this phenomenon, it remains to be clarified whether and to what extent an advanced male age can influence reproductive outcomes and thus assume prognostic relevance in the work-up of couple infertility.

It is a matter of fact that male aging could be responsible for a longer time to conception [3, 4], other than a higher risk for miscarriage [5–7], genetic abnormalities [8] and neurocognitive defects in the offspring [9, 10]. These effects could also reflect and coexist with a worsened quality of semen parameters, as a decline in semen volume, total sperm count, sperm motility and normal sperm morphology has been reported in association with advanced male age [11].

While the maternal age of 35 years has been proposed as a threshold beyond which the possibility of pregnancy is reduced and the risk of abortion and malformations in the offspring are increased, to date, no shared age cut-offs have been established that clearly define an “advanced paternal age”.

The present study aimed i) to evaluate whether and to what extent there is an association between advancing male age and worsening of seminal parameters and ii) to determine whether a threshold age can be identified above which the decline in seminal parameters becomes statistically significant.

## Methods

### Study design and population

**Fig. 1:**
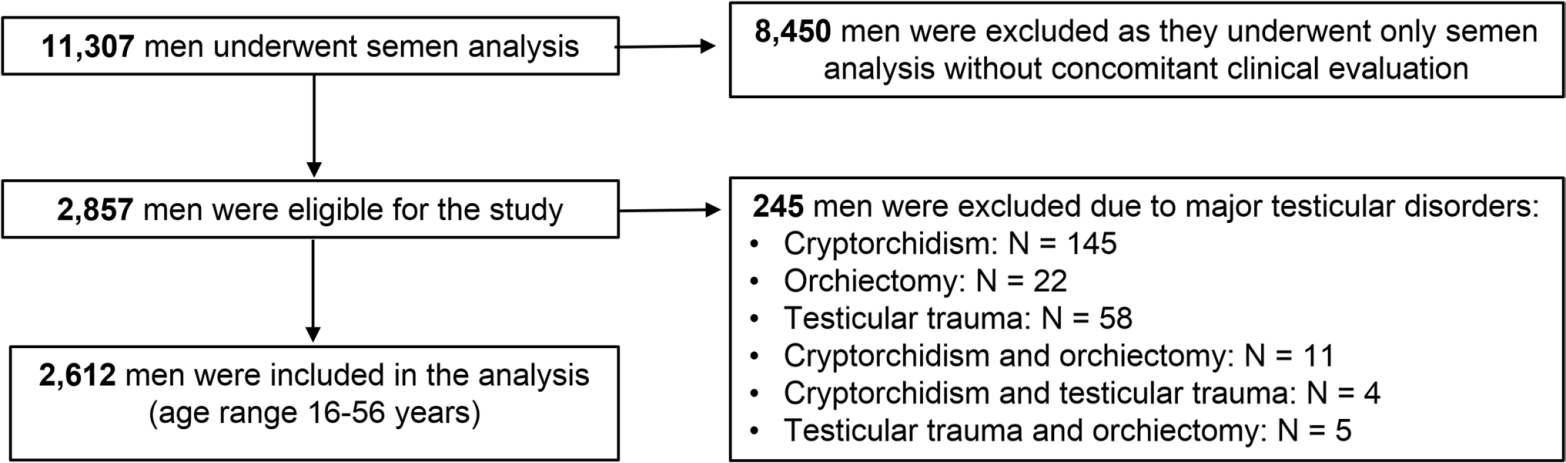
Study flow-chart

We screened 11,307 consecutive semen samples from all men who attended the Andrology Unit at the University Hospital of L’Aquila (Italy) from January 1992 to January 2022 to undergo semen analysis for any indication (Table 1). Eight thousand four hundred and fifty men were excluded because they only underwent semen analysis without a concomitant clinical evaluation: in these cases, the availability of age and seminal parameters only did not allow statistical adjustments for possible clinical confounders. Of the 2857 eligible men, we excluded those with a history of major testicular disorders (cryptorchidism, orchiectomy and/or genital trauma) that could mask any influence of age on seminal parameters. Therefore, 2612 men (age range: 16–56 years) have been included in the analyses (Fig. 1). When multiple semen analyses had been performed in the same man, only the first examination was included.

**Table 1:** Clinical and seminal variables of the study population

	Median (25th-75th centile) N = 2,612
Clinical variables	
Age (years)	36 (32–40)
Current smoking (n, %)	680 (26.0)
History of MAGI (n, %)	488 (18.7)
Varicocele (n, %)	869 (33.3)
Reason for semen analysis	
Couple infertility (n, %)	1350 (51.7)
Known varicocele (n, %)	710 (27.2)
Checking fertility potential (n, %)	528 (20.2)
Haemospermia (n, %)	24 (0.9)
Seminal variables	
Semen volume (ml)	3.0 (2.0–4.1)
Sperm concentration (10^6^ per ml)	39.0 (12.4–78.0)
Total sperm number (10^6^ per ejaculate)	104.4 (35.0–225.0)
Total motility (%)	65 (52–75)
Progressive motility (%)	49 (35–61)
Normal sperm morphology (%)	10 (4–18)
Abnormal heads (%)	81 (73–89)
Abnormal midpieces (%)	6 (3–9)
Abnormal principal pieces (%)	3 (1–7)
TMC (10^6^ per ejaculate)	66.7 (18.1–154.9)
TPMC (10^6^ per ejaculate)	48.4 (12.2–124.6)
Leukocytospermia (n, %)	109 (4.2)
Hormonal variables	
FSH (mIU/ml)^a^	7.9 (6.7–9.6)
LH (mIU/ml)^b^	4.4 (3.4–5.7)
Total testosterone (ng/ml)^b^	5.5 (4.4–6.2)
Ultrasound testicular volume	
Right (ml)	13.6 (11.0–16.0)
Left (ml)	11.9 (9.9–14.0)

*FSH* follicle-stimulating hormone, *LH* luteinizing hormone, *MAGI* male accessory gland infections, *TMC* total motile count, *TPMC* total progressive motile count

^a^Data available in 2234 men; ^b^Data available in 572 men

All the patients gave written informed consent for access to their clinical information and the use of their data, following the guidelines of the Italian Privacy Law. Clinical data were retrieved after the approval of the study by the Ethical Committee of the University of L’Aquila, protocol number 26652.

### Semen collection and analysis

All semen samples were collected into sterile containers by masturbation in a private collection room close to the laboratory, after 2–7 days of sexual abstinence. Semen analyses were performed according to the ongoing WHO-recommended procedures [12–15], although sperm concentration and progressive motility (grade a + b) were assessed in a Makler counting chamber (Sefi-Medical Instrument, Haifa, Israel), which was no longer mentioned as a suitable counting chamber in the last editions of the WHO manual [14, 15]. Sperm morphology was always assessed over time using Kruger's strict criteria after smear preparation with Papanicolau stain. Total sperm number (10^6^ per ejaculate) was calculated by multiplying the sperm concentration (× 10^6^ per ml) by the volume (ml) of the whole ejaculate. Total motile count (TMC) and total progressive motile count (TPMC) were calculated by multiplying total sperm number by total motility and progressive motility, respectively. Leukocytes were recognized as peroxidase-positive cells and leukocytospermia was defined as > 1 × 10^6^ peroxidase positive cells/mL. Semen analyses were performed over time by the same experienced biologist (G.C) undergoing periodic internal quality control. The laboratory participates in external quality assessment for semen analysis, undertaken by the UK National External Quality Assessment Service (Birmingham, UK).

### Statistical analysis

Statistical analysis was performed using R statistical software (version 4.2.2, 2022, The R Foundation for Statistical Computing, Vienna, Austria). Correlations of semen parameters with age were evaluated using the Spearman’s correlation test. Multivariable linear regression analyses on log-transformed values assessed the independent associations of age with each semen parameter, after adjustment for abstinence time, current smoking, history of male accessory gland infections (MAGI), varicocele, and the year in which semen analysis was performed. Differences in semen parameters among quartiles of increasing age of the study population were evaluated using the Wilcoxon rank-sum test or X^2^ test as appropriate, after the adjustment of α level for multiple comparisons.

## Results

The main characteristics of the study population are shown in Table 1.

**Fig. 2:**
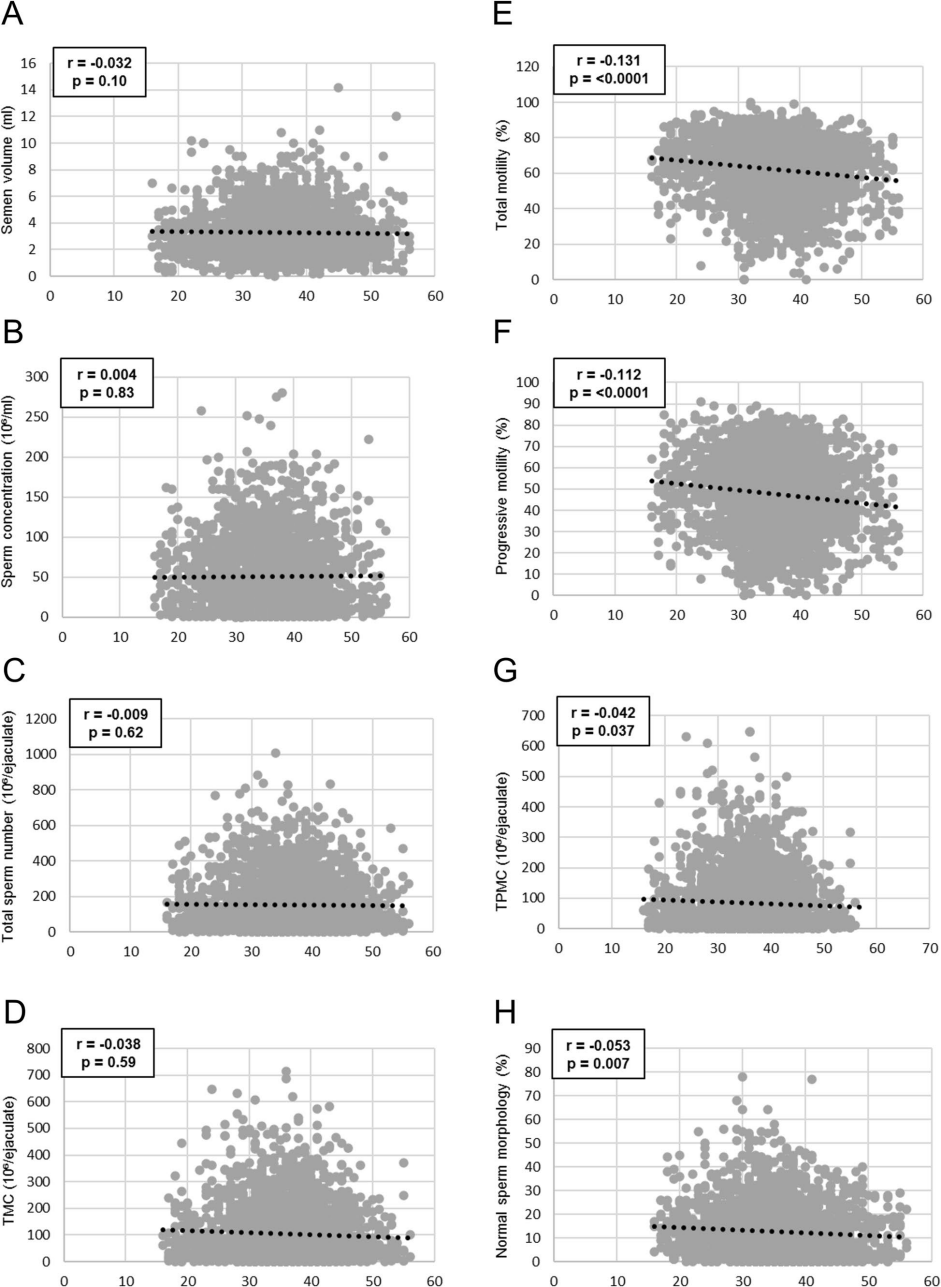
Correlations between age and semen volume (**A**), sperm concentration (**B**), total sperm number (**C**), TMC (**D**), total motility (**E**), progressive motility (**F**), TPMC (**G**), and normal sperm morphology (**H**). Spearman’s rank correlation coefficients, and *p* values are shown. Abbreviations: TMC, total motile count; TPMC, total progressive motile count

No significant correlations of age were observed with either semen volume (*r* = − 0.032, *p* = 0.10; Fig. 2A), sperm concentration (*r* = 0.004, *p* = 0.83; Fig. 2B), total sperm number (*r* = − 0.009, *p* = 0.62; Fig. 2C) or TMC (*r* = − 0.038, *p* = 0.59; Fig. 2D). On the contrary, age exhibited significant negative correlations with total motility (*r* = -0.131, *p* < 0.0001; Fig. 2E), progressive motility (*r* = − 0.112, *p* < 0.0001; Fig. 2F), TPMC (*r* = − 0.042, *p* = 0.037; Fig. 2G), and percentage of spermatozoa with normal morphology (*r* = − 0.053, *p* = 0.007; Fig. 2H). When specific pathomorphological abnormalities were considered, age did not significantly correlate with the percentage of spermatozoa with abnormal head (*r* = 0.04, *p* = 0.1), midpiece (*r* = 0.02, *p* = 0.4), or principal piece (*r* = − 0.04, *p* = 0.06).

To verify the independence of the significant associations, age-correlated semen parameters were included into multivariable linear regression models adjusted for a number of possible confounders. As shown in Table 2, after adjustment for abstinence time, current smoking, history of MAGI, and varicocele (Model I), age was negatively and significantly associated with total motility (β-coefficient: − 0.37, *p* < 0.0001), progressive motility (β-coefficient: − 0.33, *p* < 0.0001), TPMC (β-coefficient: − 1.15, *p* = 0.00016), and percentage of normal forms (β-coefficient: − 0.13, *p* = 0.0002). To rule out the possible confounding effect of an overall decline in seminal quality over time independently of individual age, linear regression analyses were further adjusted for the year in which semen analysis was performed (Model II): all the associations from Model I persisted even after the full adjustment of Model II (Table 2).

**Table 2:** Independent negative association between age and semen parameters: multivariable linear regression analyses

Parameter	Model I		Model II	
	β-coefficient (95% CI)	*p* value	β-coefficient (95% CI)	*p* value
Total motility (%)	− 0.37 (− 0.47, − 0.26)	< 0.0001	− 0.37 (− 0.47, − 0.26)	< 0.0001
Progressive motility (%)	− 0.33 (− 0.45, − 0.22)	< 0.0001	− 0.33 (− 0.44, − 0.22)	< 0.0001
TPMC (10^6^ per ejaculate)	− 1.15 (− 1.75, − 0.55)	0.00016	− 1.12 (− 1.72, − 0.52)	0.0002
Normal sperm morphology (%)	− 0.13 (− 0.19, − 0.06)	0.0002	− 0.10 (− 0.17, − 0.04)	0.002

Model I: adjusted for abstinence time, current smoking, history of male accessory gland infections (MAGI), varicocele; Model II: adjusted for Model I and year in which the semen analysis was performed. CI, confidence interval; TPMC, total progressive motile count

**Fig. 3:**
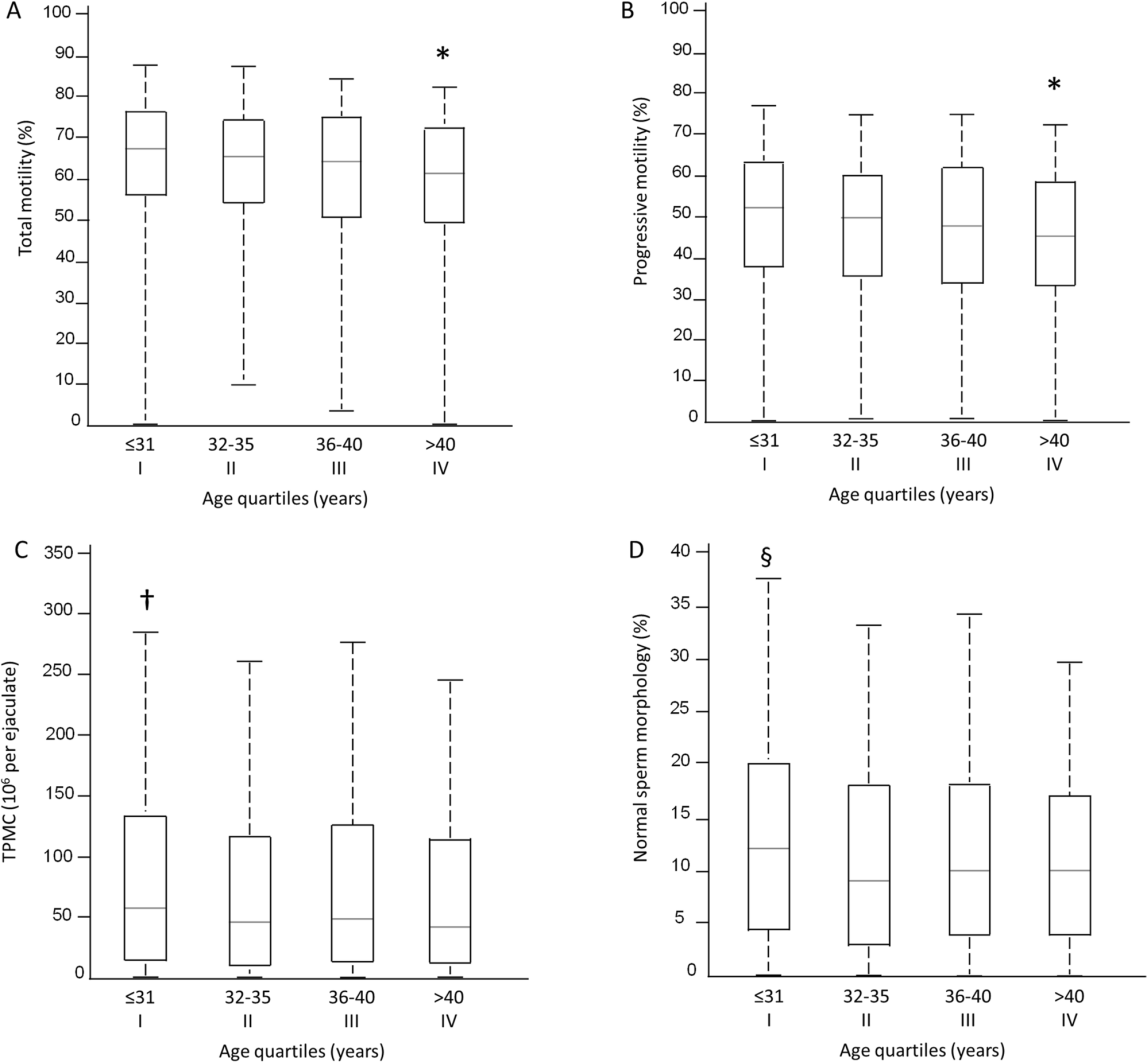
Distributions of values for total motility (**A**), progressive motility (**B**), total progressive motile count (TPMC) (**C**), and normal sperm morphology (**D**) among quartiles of increasing age. Values are medians (horizontal bars) with 25th and 75th centiles (boxes); the “whiskers” extend out to the minimum and maximum value. *p < 0.008 vs all the others; ^†^p = 0.006 vs quartile II; ^§^p < 0.008 vs quartile II and III. Based on the number of comparisons, significance was accepted for p < 0.0083

The existence of an independent negative linear association between age and most semen parameters prompted us to verify if a threshold age could be identified beyond which worsening of those parameters became statistically significant. As shown in Fig. 3, when comparisons were performed among quartiles of increasing age, the fourth quartile, corresponding to the age group above 40 years, was associated with a significant decrease in total and progressive motility. Data also pointed to an earlier decline in the TPMC and percentage of normal forms.

Among the whole study population, 311 men had azoospermia/cryptozoospermia or severe oligozoospermia (< 5 × 10^6^ sperm/ml). The prevalence of these conditions did not exhibit an upward trend from the first to the fourth quartile: the prevalence was significantly higher in the 2nd quartile (94/661, 14.2%) than in both the 1st (83/789, 10.5%, p = 0.03) and 4th quartiles (56/590, 9.5%, p = 0.01); the latter, in turn differed significantly from the 3rd quartile (78/572, 13.6%, p = 0.03).

## Discussion

Unlike the well-known role of female aging as a risk factor for fertility, the impact of advancing male age on reproductive function is still a matter of debate. The results of the present study suggest that advanced age is associated with reduced sperm motility and percentage of spermatozoa with normal morphology. Interestingly, no effect has been observed on sperm count. These results are in keeping with those from a large meta-analysis that collected information on 93,839 men from 90 studies [11]. In that study, authors documented age-associated decreases in semen volume, total motility, progressive motility, and normal morphology, as well as sperm DNA integrity. Seminal volume decrease, which was attributed to age-related testosterone decline, was not found in our series of relatively young men (median age: 36 years, range: 16–56). The negative association between age and sperm genomic integrity has been also reported in a study on 2178 men undergoing diagnostic work-up for couple infertility [16] and by a more recent systematic review of 19 articles resulting in an overall sample of 40,668 men [17]. Interestingly, consistent with our results, in the meta-analysis by Johnson and coworkers [11], sperm concentration did not decline with increasing male age. This evidence reinforces the notion of a direct impact of age on seminal parameters, regardless of the general worsening trend worldwide over time. Indeed, a common criticism of studies assessing the decline in semen quality with age is that it also declines overall over time [18]. However, this generalized phenomenon mainly affects sperm concentration [11, 18], which was found to be a seminal parameter unaffected by age. Indeed, when the analysis was restricted to cases of severe oligozoospermia to azoospermia, in our series, the prevalence of these conditions after age 40 was significantly lower than in the age groups of 36–40 and 32–35. Furthermore, in our regression analyses, significant negative associations of age with total motility, progressive motility, TPMC and normal sperm morphology persisted in the fully adjusted models also including the year in which semen analysis was performed.

Several mechanisms might explain the adverse effects of age on semen quality. As age advances, testicular parenchyma undergoes aging changes involving both the interstitial and germinal compartments [19–24]. This could contribute to explain the effects of aging on sperm motility and sperm morphology here reported, as well as the impact on sperm DNA integrity [16, 17]. A possible causative role of oxidative stress could also be suggested, as systemic circulating biological markers of oxidative stress increase with age, paralleling a depletion of antioxidant defenses [25]. Moreover, after the age of 40 years, increased seminal levels of reactive oxygen species (ROS) negatively correlated with sperm motility [26]. Of note, human spermatozoa are particularly vulnerable to oxidative stress, due to the scarce availability of cytoplasm containing antioxidant enzymes offering a first-line defense against free radicals [27]. Hence, the age-related depletion of the body antioxidant power acquires a pivotal role in the physiology of the “aged spermatozoa”. Furthermore, human sperm membrane is particularly rich in polyunsaturated fatty acids (PUFA), required in maintaining membrane fluidity and fusogenicity that are necessary properties for both acrosomal reaction and interaction with the oolemma [28]. However, PUFA are also highly sensitive to lipid peroxidation, due to the presence of carbon–hydrogen bonds with a low dissociation energy at the bisallylic methylene position [27], thus explaining the correlation between lipid peroxidation and reduced sperm motility [29, 30]. Finally, defective spermatozoa themselves are responsible for generating abnormally high quantities of ROS from mitochondrial transport chain dysfunction that may exceed cell detoxifying capacity [27]. Advancing age, indeed, is associated with a significant increase in the percentage of spermatozoa with mitochondrial depolarization, as an expression of natural senescence processes of biological structures and progressive accumulation of mutations in mitochondrial DNA [16]. The age-dependent increase in oxidative stress can not only impair the functional and genetic integrity of spermatozoa, but also Leydig cell steroidogenesis and the ability of Sertoli cells to support normal germ cell differentiation [31]. The effects of oxidative stress can be heightened during infections, and accordingly, in a study by Rolf et al. [32], total sperm count decreased significantly with advancing age only in patients with an infection of the accessory glands. Although our analyses were adjusted for history of previous MAGI, none of the participants had current MAGI, as we do not routinely perform seminal analysis in the presence of acute genital infection due to the discomfort and pain associated with seminal sample collection. This may contribute to explain the lack of significant correlation between age and sperm count in our series. Interesting evidence collected in somatic cells have shown a possible involvement of age-related epigenetic modifications too. In different tissues it has been reported that methylation levels at the cytosine-guanine island of the promoters of several genes increase with aging [33]. Similarly, a significant increase in cytosine methylation content has been also reported in ageing spermatozoa [34]. In somatic cells, the genes involved in methylation processes are mainly related to differentiation or development, which represent physiological functions that are silenced in aging. Intriguingly, it has been reported that some genes code for key proteins involved in maintaining the structural and functional integrity of mitochondria [35]. In particular, the coactivator-1 of peroxisome proliferator-activated receptor gamma (PPARγ) coordinates the activities of nuclear factors required for the expression of mitochondrial DNA genes whose products are involved in biogenesis and ROS detoxification [35]. In the mouse, the age-related methylation process is associated with a 65% reduction in the transcription of the PPARγ coactivator-1 (PGC-1) alpha gene of heart muscle, resulting in mitochondrial dysfunction and increased superoxide anion generation [36].

In couples with male partners over 40 years of age, all these possible mechanisms underlying age-related alterations in seminal quality may also result in poorer couple fertility outcomes [3] and increased risk of miscarriage [37]. In a study by Hassa and Killick [38], involving 2,112 couples, advancing men’s age was associated with significantly rising time to pregnancy (TTP) and declining conception rates, especially above the age of 45 years, even when analysis was restricted to partners of young women. In autologous oocyte cycles, a paternal age threshold of 40 years appears to be associated with significantly lower pregnancy and live birth rates [39]. Actually, in the model of assisted reproductive technology, a not negligible role is played by the maternal age and therefore oocyte quality. After controlling for these female variables by using the donor oocyte cycles model, the association of paternal age with worse outcomes of assisted reproductive technology was resized, strongly suggesting that it is the combination of both advanced paternal and maternal age that substantially lowers success chances by reducing the compensatory capacity that partners exert on each other's reproductive potential [39].

This study has some limitations. First, the change of recommendations for seminal analysis among different editions of the WHO manual over time could hinder the comparability of seminal analyses. However, this limitation is counteracted by the fact that, as mentioned above, not only have Kruger's strict criteria for the assessment of morphology always been in use in our laboratory, but sperm concentration and motility have always been assessed with the Makler counting chamber by the same experienced biologist (G.C) undergoing periodic internal quality control. Furthermore, in this study, seminal data were reported and analyzed as continuous variables without including dichotomization thresholds in percentiles, thus overcoming an obvious major difference in the reporting of seminal examination according to the WHO 2010 [14] and 2021 [15] recommendations compared with previous editions of the manual. Second, our results may not extend to the general population as they were produced in men assessed in a clinical setting devoted to the management of andrological disorders. Third, neither genomic integrity nor oxidative stress was assessed in the present study. Such assays could have provided interesting insights into possible mechanisms underlying the age-related decline in semen quality. However, sperm DNA fragmentation assay and ROS testing are included among the extended and advanced examinations, respectively [15]. Therefore, since these tests are not expected to be routinely performed as part of the standard semen analysis, the unavailability of these data in a study with a retrospective design is not surprising. Similarly, seminal biochemical parameters were not available, as they were not part of routine seminal analysis. Such data could have provided interesting information since the composition of seminal plasma could change with aging of the male accessory genital glands and, in turn, abnormal seminal plasma composition could affect seminal quality. Fourth, seminal parameters are subject to large spontaneous intraindividual variability [40]; therefore, the analyses should include repeated seminal assessments for each subject. This limitation should also be considered intrinsic to any retrospective study, and, in any case, it can be partially counteracted by the large sample size of our series. Furthermore, although analyses were adjusted for a number of clinical confounders, as in any retrospective study, we cannot exclude that residual unmeasured confounding has influenced the associations under investigation. In particular, analyses were not adjusted for systemic comorbidities, including obesity, nor for medications, nor for lifestyle habits, such as drug abuse: unavailability of complete and detailed information in this regard for many participants would have reduced the sample size at multivariable regression models. Last but not least, it should be considered that although age was negatively associated with semen quality, this does not necessarily mean that advancing age leads to reduced couple fertility for a variety of reasons: 1. Although from the age of 40 years some of the standard seminal parameters worsen significantly compared to earlier age groups, the values remain largely within the fertile range of the WHO reference population [15]; 2. Seminal parameters represent a surrogate marker of fertility with much less prognostic significance than clinically relevant fertility end-points such as TTP, pregnancy rate and live birth rate; 3. The impact of a poor semen quality on couple reproductive outcomes can be largely mitigated and counterbalanced by an optimal female fertility potential [39]. Nevertheless, seminal examination remains the first-level assessment in the diagnostic workup of the infertile couple and the basis for identifying a male factor of infertility.

In conclusion, advancing male age is associated with a decrease in the proportion of motile and morphologically normal spermatozoa, independent of a number of clinical confounders and with greater evidence from the age above 40 years. Although the mechanisms of these associations largely remain to be elucidated, a key role may be played by oxidative stress that progressively increases with age. This evidence could have clinical implications: healthcare providers should be aware that worsening seminal quality with age can impair the male partner's ability to compensate for a female subfertility condition resulting in an overall weakening of the couple’s reproductive potential. In this view, the inclusion of analyses for sperm oxidative damage (e.g. DNA integrity), as well as the use of pharmacological and lifestyle “antioxidant” strategies could be suggested in infertility workup of men over 40 years of age.

## Data Availability

The data that support the findings of this study are available from the corresponding author (A.B.), upon reasonable request.
